# Humeral Aneurysmal Bone Cyst in a Cat with Sequential Computed Tomographic Findings

**DOI:** 10.3390/vetsci9110594

**Published:** 2022-10-27

**Authors:** Kihoon Kim, Hyungjoon Kim, Hyosung Kim, Yeonhea Lee, Jaehwan Kim, Sunhee Do, Hwiyool Kim

**Affiliations:** 1 Department of Surgery, College of Veterinary Medicine, Konkuk University, Seoul 05029, Korea; 2 Baeksan Feline Medical Center, Seoul 06223, Korea; 3 Department of Clinical Pathology, College of Veterinary Medicine, Konkuk University, Seoul 05029, Korea; 4 Department of Veterinary Medical Imaging, College of Veterinary Medicine, Konkuk University, Seoul 05029, Korea

**Keywords:** aneurysmal bone cyst, cat, CT, humerus

## Abstract

**Simple Summary:**

This case report describes a case of an aneurysmal bone cyst of the humerus in a cat with sequential computed tomography findings acquired 4 months after the first visit. An aneurysmal bone cyst is a tumor-like lesion with massive bone destruction. It is a benign, osteolytic, blood-containing bone lesion that grows rapidly. It is thus often misdiagnosed as a malignant tumor. The most practical treatment in advanced cases is amputation of the affected bone.

**Abstract:**

A 7-year-old spayed female domestic shorthair cat presented with a swollen right forelimb and mild lameness. On physical examination, the mass was palpable in the right humeral region, and the cat exhibited pain on palpation. Radiography revealed an expansile osteolytic lesion at the proximal end of the right humerus. Computed tomography (CT) revealed an expansile bony mass on the proximal end of the right humerus and a mild periosteal reaction around the acromion of the scapula. Amputation of the right forelimb, including the scapula and removal of the axillary lymph node, were strongly recommended to the owner, but were declined. Four months after the initial presentation, the cat presented with a dramatically swollen right forelimb and progressive lameness. CT was performed again. In addition to osteolytic changes in the mass, vascular development had occurred at the cranioproximal region. The right forelimb, including the scapula and ipsilateral lymph nodes, was removed. The cat died during the postoperative recovery period. Based on clinical, diagnostic imaging, and histological findings, the final diagnosis was aneurysmal bone cyst. To the best of our knowledge, this is the first case of an aneurysmal bone cyst in the humerus of a cat.

## 1. Introduction

Aneurysmal bone cysts (ABCs) are benign, osteolytic, and blood-containing lesions of the bone bulging the overlying cortex [1,2]. Blood-filled cavities of ABCs are separated by thin-walled connective tissue septa [3,4]. Owing to the rapid growth of these tumor-like lesions and the resulting massive bone destruction, they are often misdiagnosed as malignant tumors [2].

ABCs are frequently identified in human medicine, with the spine, pelvis, and long bones being most commonly involved [5]. In veterinary medicine, although recognition of ABC’s is increasing, they are still rare in animals such as horses, cattle, dogs, and cats [5]. Two cases of pelvic ABC have been reported in cats [3,6]. Scapula, sacrum, and caudal vertebrae involvement have also been reported in cats [7,8].

The precise etiology and pathogenesis of this condition remain unclear [1,5]. Although various imaging modalities are informative for diagnosis, definitive diagnosis requires histopathological examination [5]. Common clinical signs include pain and swelling due to bone destruction and swelling of the surrounding soft tissues [5,7]. Functional impairments have also occasionally been reported [5]. Cyst wall curettage, a standard treatment in human medicine, is often combined with various adjuvant therapies, such as cryotherapy, sclerotherapy, argon beam coagulation, or bone cement. A standard treatment for cats has not yet been determined [3]. However, the most practical treatment in advanced cases is amputation of the affected bone [2].

To the best of our knowledge, this is the first detailed report describing a humeral aneurysmal bone cyst in a cat with sequential computed tomographic (CT) findings obtained 4 months after the first visit.

## 2. Case Presentation

A 7-year-old spayed female domestic shorthair cat, weighing 5.54 kg (body condition score 5/9), was presented at the Baeksan Feline Medical Center for evaluation of a swollen right forelimb and mild lameness. The owner reported that swelling of the right forelimb was identified suddenly 2 month prior to presentation; lameness was also observed at that time. The patient had no history of trauma. On physical examination at presentation, the cat was unremarkable in gross appearance, except for swelling of the right humerus and scapular region. Orthopedic examination revealed a mild pain reaction upon palpation around the mass.

The complete blood count was within normal limits [red blood cell (RBC) 9.81 M/uL, reference range (RR) 6.54–12.2; hematocrit (HCT) 55.7%, RR 30.0–52.3; hemoglobin (HGB) 15.6 G/dL, RR 9.8–16.2]. However, serum chemical analysis revealed an increased alkaline phosphatase (ALKP, 173 U/L, RR 14–111) level.

**Figure 1:**
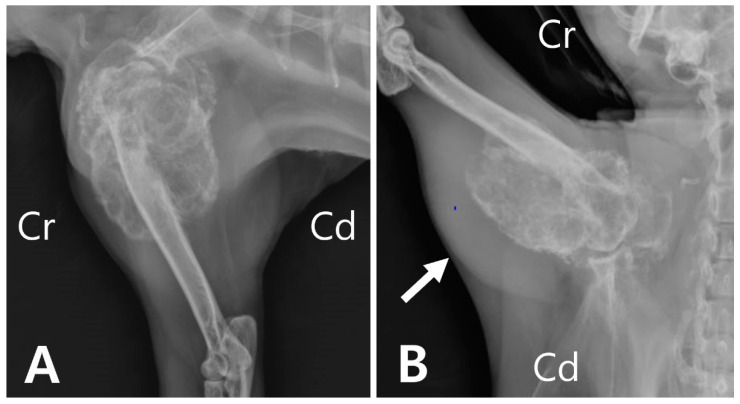
(**A**) A large expansile, lobulated mass of mixed osteolytic and osteoproliferative areas involving the proximal humerus. (**B**) Note marked soft tissue swelling around the lesion (arrow) of the humerus.

Radiography revealed an extensive area of expansile and lytic bone lesions of the right proximal humerus. The thin-walled bony mass was compartmentalized by trabeculations traversing an area of radiolucency, resulting in a “soap bubble” appearance (Figure 1A). Soft tissue swelling was also observed overlying the region adjacent to the humeral lesion (Figure 1B).

**Figure 2:**
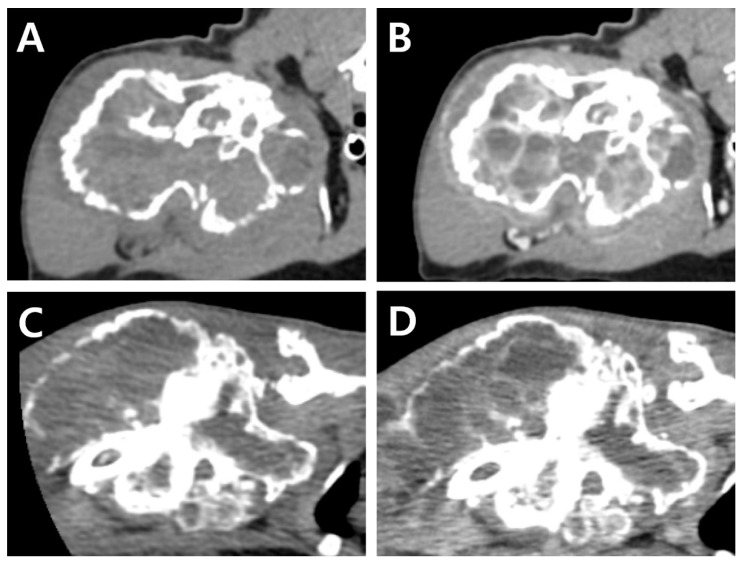
CT images at initial presentation (**A**,**B**). Pre-contrast image (**A**), post-contrast image showing contrast-enhancement of the surrounding tissues and vessels within the mass (**B**). CT Images at the second presentation (4 months after initial presentation) (**C**,**D**). Pre-contrast image (**C**), and post-contrast image (**D**). Note, regardless of the change in the size of the mass, fluid attenuating cystic lesion with rim enhancement of margin and internal septa are consistently identified.

CT (Revolution^®^; GE Healthcare, Waukesha, WI, US) images were acquired before and after intravenous injection of the contrast medium (Omnipaque 300; GE Healthcare, Milwaukee, WI, USA). Consequently, a lytic expansile hypoattenuating bony mass (3.8 cm × 3.6 cm × 6.3 cm, Figure 2A) was identified between the humeral head and the mid-diaphysis. Severe cortical and medullary destruction, a sclerotic transition zone, and enriched vascularity were also noted around and inside the bony mass. Soft tissue blood density was observed in the lesion. Moreover, a periosteal reaction was observed around the acromion and glenoid cavity, with mild cortical destruction in the scapula. A post-contrast CT scan revealed contrast enhancement of the soft tissue adjacent to the mass. Homogeneous fluid attenuating signal intensity of cystic content and thin rim enhancement of margin and internal septa were also identified. (Figure 2B). The axillary lymph node was slightly enlarged (1.7 cm × 1.5 cm × 0.68 cm).

Based on radiological and CT findings, limb-sparing surgery was not considered suitable because of the large size of the mass. Moreover, the thin cortex of the bone was thought to be vulnerable to multiple biopsies, increasing the possibility of fractures. Therefore, amputation of the right forelimb, including the right scapula and axillary lymph node, was recommended, but was declined by the owner.

Four months after the first visit, the cat was returned to the hospital because of a more swollen right forelimb and worsening lameness. On gross appearance, the proximal region of the humerus was enlarged compared to its size at the time of initial presentation. On physical examination, more limited movement of the shoulder joint was identified than in the initial presentation.

**Figure 3:**
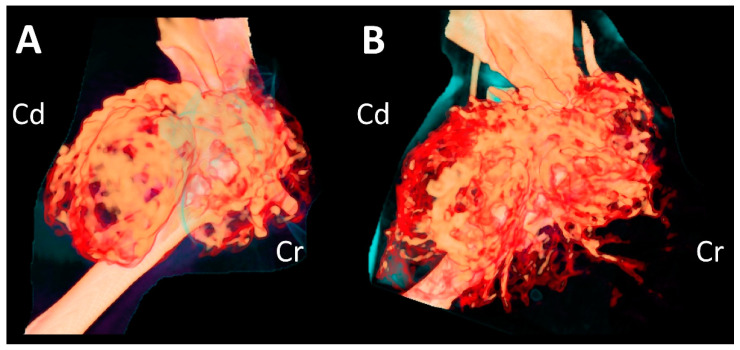
Three-dimensional volume-rendered images of the humeral mass at initial presentation (**A**) and second presentation (**B**), showing increased vascular proliferation around the mass.

A complete blood count revealed severe anemia (hematocrit, 20.0%, reference range [RR] 30.3–52.3%). Four months after the initial presentation, a CT examination was performed again prior to the surgery (Figure 2C,D). Compared to the CT findings on initial examination, the mass had expanded dramatically in various planes. The overall thickness of the cortex of the mass had decreased, showing greater destruction of the medulla. Cortical destruction of the acromion and glenoid cavity was also identified, showing a more irregular surface due to the periosteal reaction. On three-dimensional volume-rendered images, osteolytic changes in the cranio-proximal region were more prominent, resulting in poor lesion margination at the time of the second presentation. In addition to the osteolytic changes, spiculated osteoid and areas of marked contrast enhancement were clearly visible, suggesting vascular proliferation in the surrounding soft tissue (Figure 3). The ipsilateral axillary lymph node had enlarged (1.9 cm × 1.75 cm × 0.79 cm) since the previous examination.

**Figure 4:**
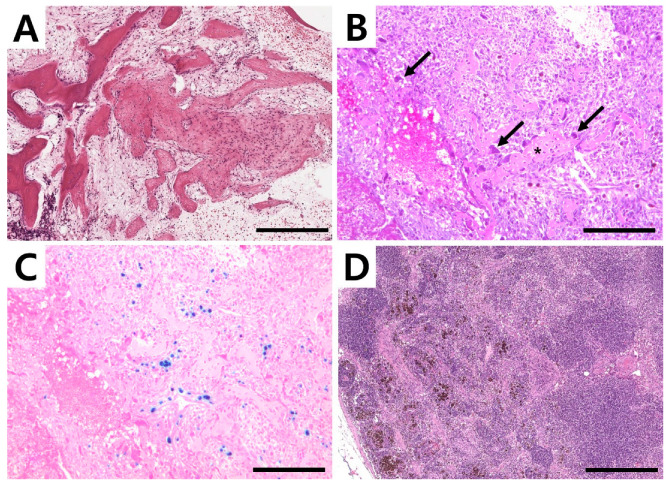
Histological appearance of the lesion. (**A**) Areas of fibrous tissue proliferation and reactive bone (H&E, scale bar = 500 µm). (**B**) Cavernous spaces delimited by septa of connective tissue with the presence of giant multinucleated cells. Note bone spicules (asterisk) with osteoblastic activity (white arrow) and presence of osteoclasts (black arrow) (H&E, scale bar = 200 µm). (**C**) Macrophages phagocytosing hemosiderin (Prussian blue, scale bar = 200 µm). (H&E, scale bar = 200 µm). (**D**) Lymph node with hemosiderin-laden macrophage (H&E, scale bar = 500 µm).

Amputation of the right forelimb was again strongly recommended, and the owner elected the surgery this time. The right forelimb, scapula, and ipsilateral axillary lymph nodes were removed. Macroscopically, the lesion consisted of a honeycomb structure that contained blood. For histopathological evaluation, tissue samples, including the humeral mass and lymph node, were surgically removed and fixed in 10% neutral-buffered formalin. The bone segment was further decalcified by using a decalcifying solution (Sigma-Aldrich, St. Louis, MO, USA) following the manufacturer’s instruction. The tissues were embedded in paraffin, cut into 4 µm-thick sections, and the sections were stained with hematoxylin and eosin (H&E). Histopathological examination of the lesion tissue revealed a larger area of loose fibro-osseous proliferation and trabecular woven bone, most consistent with an aneurysmal bone cyst (Figure 4A). The bone composing the cyst had no characters of atypia or mitosis. Blood-filled spaces were surrounded by moderately cellular spindle cell proliferation with minimal atypia, edema fluid, perivascular cluster of lymphocytes, and plasma cells. Cavernous spaces were delimited by septa of connective tissue with the presence of giant multinucleated cells, most likely osteoclasts. Bone spicules and trabeculae with osteoblastic activity were identified (Figure 4B) together with hemosiderin-laden macrophages (Figure 4C). The subcapsular and medullary sinuses of the right axillary lymph node were expanded by foamy and hemosiderin-laden macrophages (Figure 4D), red blood cells, neutrophils, and a small amount of cellular debris. Lymphoid follicles were abundant and hyperplastic with retained polarity, indicating lymphoid hyperplasia and reactivity associated with drainage from the primary site of hemorrhage and inflammation. No neoplastic cells were identified.

Based on radiographic, histological, and clinical findings, a final diagnosis of an aneurysmal bone cyst was made. Follow-up after surgery could not be performed because the patient died during the postoperative recovery period. Non-cardiogenic pulmonary edema was strongly suspected as the main cause of death; however, the precise cause was not identified because the owner declined necropsy.

## 3. Discussion

The precise etiology of ABC is undetermined, and the following theories have been proposed: a regional disruption of the bone marrow vasculature leading to arteriovenous shunting, hematoma or subperiosteal hemorrhage secondary to trauma, or a transformation of giant cell tumor [2,9,10]. A disparity in blood circulation between the arterial and venous flow of a lesion, causing an incomplete venous obstruction, has been previously reported for the pathogenesis of ABC [2].

ALKP is an omnipresent enzyme found in many tissues but is mainly present in the liver, kidney, placenta, and bone [11]. In the musculoskeletal system, ALKP is released by osteoblasts and is thought to play a role in new bone formation [12]. In veterinary medicine, although the correlation between ALKP and ABC is not yet well understood, it is assumed that the increase in ALKP levels is the result of prominent osteolytic changes in ABC [4,13]. In this case report, increased ALKP levels were identified, which may have resulted from reactive bone formation.

Radiographically, ABCs have a typical characteristic appearance, revealing expansile osteolytic lesions that are relatively eccentric to the long axis of the bone [14,15]. However, owing to its locally destructive nature, it is difficult or often impossible to differentiate it from other malignant tumors such as telangiectatic osteosarcomas. Both ABCs and telangiectatic osteosarcomas show rapid growth, affecting the medullary and cortical bones. Thus, histopathological examination is necessary to differentiate ABCs from telangiectatic osteosarcomas or unicameral bone cysts [3].

Ultrasonographic findings of ABC include swirling echogenic fluid within the cavitary lesion, as the blood within the ABC is unclotted and in constant motion [2,14]. This ultrasonographic feature might be helpful in differentiating ABC from other malignant tumors, except telangiectatic osteosarcoma [14]. In addition to the freely flowing blood within the cavity, Dowdle et al. observed a lower resistivity index (RI) in affected arteries than in normal arteries using ultrasonography. They speculated that this phenomenon was due to an arteriovenous shunt permitting blood to bypass the capillary bed [2].

CT examination may also be used for the diagnosis of ABC. Previously, Birincioglu et al.’s without contrast study, revealed an expansile hypodense lesion in the medullary area of the right humerus in a dog [4]. In addition to diagnostic tools, CT has also been used for treatment. Uhlgorn et al. used CT for surgical planning [3]. A combination of CT-guided cementoplasty and radiation therapy in a cat has also been reported [16]. To the best of our knowledge, this is the first reported veterinary case describing sequential CT findings over 4 months in ABC. At initial presentation, on CT examination, soft tissue blood density was observed inside the mass, suggesting that it was not an osteosarcoma. Moreover, cavity-like vessels were observed on post-contrast CT, which is a significant finding for the presumptive diagnosis of ABC. However, according to the second CT, vascular formation outside the mass had dramatically increased, suggesting osseous hemangioma or hemangiosarcoma as the possible origin of the mass. Thus, since differential diagnosis based on CT findings of ABC over time may differ, the diagnosis of ABC should be based on various clinical findings, including history, various imaging findings, and histopathological findings in addition to CT findings.

To make a final diagnosis of ABC, multiple biopsies from various areas of the mass are essential [2,14]. However, the requirement for multiple biopsies may be challenging in cases where there is an extremely thin bone cortex. In such cases, pathological fractures can occur around the biopsy site [14]. Thus, in the current case, amputation was considered rather than multiple biopsies for diagnosis.

ABCs generally do not metastasize; however, in human medicine, metastasis to the lungs, liver, and kidneys has been reported [4]. In the present case, metastasis to other organs was not identified in any of the CT examinations. Whole-body bone scintigraphy can be used to exclude distant metastasis [2,4].

A recommended treatment for ABC in veterinary medicine has not yet been established. Many treatments, including bone grafting, curettage, amputation, and adjuvant therapy such as radiation, have been described in human literature [2,4,5,17]. Cementation, sclerotherapy or segmental osteotomy have been reported in companion animals [2,14,16]. Curettage and cancellous bone grafts have also been reported to have poor prognoses [17,18]. Patients affected by ABC often present with a late phase of the disease; thus, the affected bone shows a large lesion [2]. Vascularized autogenous bone grafts have been used in veterinary medicine [2]. Due to the technical difficulty of vascular anastomosis, the time-consuming nature of the procedure, and expensive equipment required, it is not commonly applied in veterinary medicine [2]. In this case, amputation was performed because of the extensive size of the mass and the low possibility of the affected forelimb returning to normal function. However, a limitation of this study is that the cat survived only a few days after surgery. The outcome of amputation for ABC could therefore not be determined. Although the standard treatment for ABC in veterinary medicine has not been determined, amputation of the affected limb is reported to have a good prognosis [1,7]. Thus, the short survival time of this cat could be associated with poor preoperative condition rather than the surgical procedure itself.

## 4. Conclusions

This report describes ABC of the humerus in a cat with sequential CT findings over time. Since ABC of the humerus has not been reported in cats thus far, ABC should be considered as a differential diagnosis for cats with humeral masses. Moreover, CT findings obtained 4 months apart could be valuable for the understanding of the progression of ABC.

## Data Availability

Not applicable.
